# The Role of Macrophages in Aortic Dissection

**DOI:** 10.3389/fphys.2020.00054

**Published:** 2020-02-05

**Authors:** Xinhao Wang, Hongpeng Zhang, Long Cao, Yuan He, Airong Ma, Wei Guo

**Affiliations:** ^1^Department of Vascular and Endovascular Surgery, The First Medical Center of Chinese PLA General Hospital, Beijing, China; ^2^Department of General Surgery, PLA No. 983 Hospital, Tianjin, China; ^3^Department of Obstetrics, Zibo Central Hospital, Zibo, China

**Keywords:** aortic dissection, macrophage, inflammation, Ang II, aortic wall

## Abstract

Aortic dissection (AD) is a fatal disease that accounts for a large proportion of aortic-related deaths and has an incidence of about 3–4 per 100,000 individuals every year. Recent studies have found that inflammation plays an important role in the development of AD, and that macrophages are the hub of inflammation in the aortic wall. Aortic samples from AD patients reveal a large amount of macrophage infiltration. The sites of macrophage infiltration and activity vary throughout the different stages of AD, with involvement even in the tissue repair phase of AD. Angiotensin II has been shown to be an important factor in the stimulation of macrophage activity. Stimulated macrophages can secrete metalloproteinases, inflammatory factors and other substances to cause matrix destruction, smooth muscle cell apoptosis, neovascularization and more, all of which destroy the aortic wall structure. At the same time, there are a number of factors that regulate macrophages to reduce the formation of AD and induce the repair of torn aortic tissues. The aim of this review is to take a close look at the roles of macrophages throughout the course of AD disease.

## Introduction

**FIGURE 1:**
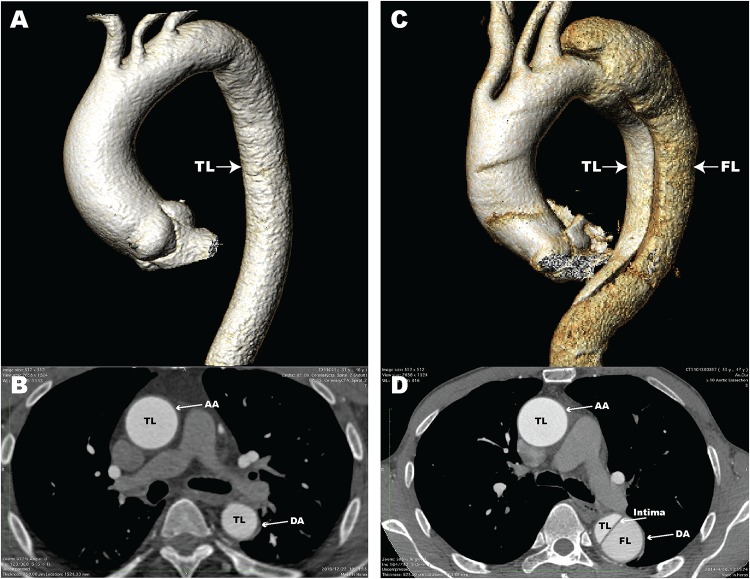
Computed tomography angiography (CTA) images of a normal aorta and an aortic dissection (AD). **(A)** 3D reconstructed CTA image of a normal aorta; **(B)** Axial CTA image of a normal aorta with blood flow only in the true lumen (TL); **(C)** 3D reconstructed CTA image from a patient with AD; **(D)** Axial CTA image of AD with blood flow in both the true lumen and false lumen (FL). The aortic intima is located between the TL and FL. AA, ascending aorta; DA, descending aorta.

Aortic dissection (AD) is a fatal disease that accounts for a large proportion of aortic-related deaths (Hiratzka et al., 2010). Epidemiological surveys have shown that the incidence of thoracic AD is 3–4 per 100,000 individuals per year (Olsson et al., 2006; Kochanek et al., 2011). AD is defined as blood flow that enters the aortic media through intimal tears, followed by formation of a true lumen (TL) and a false lumen (FL) with or without communication (Han et al., 2018). Computed tomography angiography (CTA) images of normal aorta and AD in patients from our center are shown in Figure 1. Clinically, AD can cause a series of serious complications, including aortic rupture and visceral ischemia. The majority of untreated patients with extended Stanford type A AD involving the ascending aorta die within 2 weeks (Chen et al., 1997). Clinical treatment options for AD include optimal medical treatment, aortic replacement, and thoracic endovascular aorta repair (Jia et al., 2013). However, the mortality rate for patients after treatment for AD remains high (Khayat et al., 2018). Surgical mortality for AD ranges from 10 to 35%, even at experienced medical centers (Nienaber and Clough, 2015). Unfortunately, because of its sudden and unpredictable nature, little is known about the pathological and molecular events that occur before and after the onset of AD, thus it is critical that we clarify the pathogenesis.

While ascending ADs are clearly linked to inherited connective tissue diseases (Isselbacher, 2005), most ADs occur in the sixth decade of life in hypertensive populations without genetic susceptibility (Hagan et al., 2000). The main histological finding related to arterial wall weakening in AD is medial degeneration, which consists of profound degradation of the extracellular matrix (ECM) involving smooth muscle cell (SMC) depletion (Ramanath et al., 2009; Wang et al., 2012; An et al., 2017), elastic fiber fragmentation (Nakashima, 2010; Pratt and Curci, 2010; Roberts et al., 2011) and collagen degradation (Wang et al., 2006; Ren et al., 2014; Xu K. et al., 2018). However, many studies have demonstrated that the formation of AD is associated with aortic wall inflammation (Wu et al., 2013; Cifani et al., 2015; Niinimak et al., 2018). A recent study in a murine model demonstrated that indomethacin reduces the rates of AD by inhibiting macrophage accumulation (Tomida et al., 2019). Macrophages, which have both inflammatory and anti-inflammatory effects, are involved in the development of AD (Andreata et al., 2018; Visona et al., 2018; Xu Y. et al., 2018; Ye et al., 2018) as well as the complications of AD, such as acute AD-associated lung injury (Wu et al., 2016). Our laboratory focuses on identifying the factors that cause the accumulation of macrophages in the aortic wall, and how the effects of macrophages on the structure of the aortic wall lead to AD. Here, we review current knowledge about the role of macrophages in the formation of AD, including their upstream regulators and downstream effectors.

## Evidence of Macrophage Participation in the Formation of Ad

Macrophages play a crucial role in aortic wall inflammation, and also are involved in AD. In an AD mouse model, T lymphocytes, macrophages and neutrophils simultaneously infiltrate the aorta when AD occurs, with macrophages being the most abundant cell type (Li et al., 2019). There is significant infiltration of macrophages into the tear section in AD patients (Xu Y. et al., 2018; Ye et al., 2018). Macrophage infiltration may be more severe in AD than in aortic aneurysm (Pisano et al., 2017). Half of the macrophages in the torn part of the AD mice model are BM-derived and half are non-BM-derived (Zou et al., 2019). Studies in AD cases classified as Stanford type A have shown that the aggregation of macrophages in the aortic media is critical for early AD formation (Cifani et al., 2015; Niinimak et al., 2018). However, the infiltration activity and location of macrophages vary among the different stages of AD. Overall, macrophages first accumulate in the aortic adventitia and infiltrate the media to promote a local inflammatory response after dissection. Compared with chronic AD (CAD), acute AD (AAD) is characterized by more severe inflammation in the media and adventitia, and more macrophage infiltration (Wu et al., 2013). Macrophages (CD68^+^) in the acute phase are concentrated in the hematoma, and the intima and media adjacent to the hematoma. In the subacute and early organizing phases, macrophages are mainly concentrated in the peripheral adipose tissue. The degree of macrophage infiltration is related to the repair process of the clot and adjacent vessel walls, and occurs in a time-dependent manner (Visona et al., 2018). This suggests that macrophages may be involved in not only AD formation but also AD remodeling. In fact, M1 macrophages are known to promote inflammation, while M2 macrophages eliminate inflammation and secrete and stabilize matrix components (Duffield, 2003). In the aortic repair stage, macrophages in the peripheral adipose tissue aggregate to participate in vascular wall repair.

Macrophages are also associated with some complications of AD, including FL rupture (Xu and Burke, 2013) and acute lung injury (Wu et al., 2016, 2017a,b). Therefore, macrophages play a vital role in AD.

## Angiotensin Ii Regulates Macrophages to Cause the Onset of Ad

Studies have shown that Angiotensin II (Ang II) contributes to the development of AD in humans and experimental animals (Daugherty et al., 2000). Ang II is the main effector peptide of the renin-angiotensin system. It can induce vasoconstriction, hypertrophy, and extracellular remodeling through the Ang II type 1 receptor (AT1R) (Dimmeler et al., 1997; Schluter and Wenzel, 2008). The regulatory effect of Ang II on macrophages is also crucial in AD. During the onset of AD, Ang II can promote the infiltration of macrophages from the aortic adventitia to the media through a pathogenic pathway involving serum lipid composition (Tanaka et al., 2018). Weighted gene co-expression network analysis has identified FKBP11 as a key regulator in Ang II-induced AD. FKBP11 operates through a nuclear factor-kB-dependent pathway, and is hypothesized to promote macrophage infiltration and M1 differentiation (Wang et al., 2017). Ang II also promotes the infiltration of macrophages and the secretion of matrix metalloproteinases (MMPs) via the axis of Kruppel-like factor 6 and granulocyte macrophage-colony-stimulating factor (GM-CSF) (Son et al., 2015), as well as a disintegrin and metalloproteinase with thrombospondin motifs 1 (ADAMTS-1) (Gao et al., 2016), to cause local inflammation and tissue destruction. In addition, GM-CSF triggers the separation of the aortic wall (Son et al., 2015).

Several factors are known to enhance the regulation of macrophages by Ang II in AD. First, the rs12455792 variant of the SMAD4 gene has been shown to increase macrophage recruitment and the M1 type inflammatory response via activation of transforming growth factor-β signaling, and also to promote the vascular degeneration and pathological progress of thoracic AD (Wang Y. et al., 2018). Second, the T helper cell (Th)17-interleukin (IL)-17 axis, which is regulated by IL-6-signal transducer and activator of transcription (STAT)3 signaling, is a key regulator of Ang II-induced vascular inflammation. It works upstream of the macrophage recruitment induced by Ang II (Ju et al., 2013). Third, thrombospondin 1, which increases mRNA expression inducible nitric oxide synthase (*iNOS*) in macrophages treated with Ang II, might participate in AD by boosting differentiation of M1 macrophages and apoptosis of SMCs (Zeng et al., 2019). In addition, vascular SMC-specific E-prostanoid receptor 4 deletion exacerbates Ang II-induced AD by increasing macrophage infiltration (Xu et al., 2019). Also, the study by Ju et al. (2013) indicated that the acceleration of AD in Ang II-infused mice had nothing to do with the Ang II-associated increase in blood pressure (Ju et al., 2013). This suggests that simply lowering blood pressure without also reducing the amount of Ang II in serum might adversely affect efforts to control the incidence of AD.

## Macrophages Secrete Multiple Factors Involved in Ad Formation

Studies have shown that macrophages secrete a variety of factors associated with AD, including metalloproteinases, ILs, vascular endothelial growth factor (VEGF), and others. These factors cause further macrophage recruitment, vascular SMC apoptosis and elastic fiber degradation, ultimately leading to AD.

### Metalloproteinases That Play an Important Role in AD

Metalloproteinases include an extensive zinc-dependent collagenases and elastases, which belong to the superfamily of metzincins, and are closely related to inflammation and tissue damage. Each metalloproteinase has multiple expression profiles that are unique to different tissue types and occur during inflammation, each exerting specific functions (Visse and Nagase, 2003). Several are secreted by macrophages and participate in the formation of AD. The most important of these are the MMPs and the ADAMTS enzymes.

Matrix metalloproteinases are able to regulate inflammation and tissue remodeling when in balance with tissue inhibitors of metalloproteinases (TIMPs), and this regulation plays an important role in AD. An imbalance between MMPs and their TIMPs can cause AD artery wall remodeling and degradation of the exogenous matrix (Cifani et al., 2015). Studies have shown that a variety of the MMPs that are elevated in AD – namely MMP-8, MMP-9 and MMP-12 – are mainly secreted by macrophages. Among these, MMP-9 is also closely related to acute lung injury caused by AD (Wu et al., 2017b), and targeted depletion of macrophages suppresses AD together with spatial regulation of MMP-9 in the aorta (Li et al., 2019). While MMP-3 itself is not secreted by macrophages, it is also significantly elevated in AD patients and can activate MMP-8 secreted by macrophages. This amplification cascade induces a widespread degradation of the aortic wall (Gronski et al., 1997). At the same time, studies have shown that MMP-12 is a biomarker for some types of AD, and that it can even play a role in identifying patients at greater risk of AD (Proietta et al., 2014; Cifani et al., 2015; Liu et al., 2018). Taken together, these data on MMPs support the importance of macrophages in the development of AD.

The ADAMTS enzymes play important roles in many vascular diseases and, similar to MMPs, are linked to tissue destruction and inflammation. The ADAMTS family comprise key extracellular metalloproteinases involved in ECM turnover (Gao et al., 2016; Ren et al., 2017; Wang S. et al., 2018). ADAMTS-1 and ADAMTS-4 were found to be related to AD, with increased expression levels in macrophages (Ren et al., 2013). Increased levels of circulating ADAMTS-1 have been correlated with the presence of accumulated ADAMTS-1-positive macrophages in aortic tissues in AAD patients (Ren et al., 2013; Gao et al., 2016; Wang S. et al., 2018). In experiments on elderly mice fed with Ang II, the incidence of AD was 42%, and the macrophages and neutrophils that infiltrated the aortic media were found to have elevated ADAMTS-1. The aortic tissue from AAD mice exhibited enhanced expression of ADAMTS-1, and ADAMTS1-immunoreactive macrophages infiltrated the intima, media and adventitia in dissected aortic walls (Gao et al., 2016). The incidence and rupture rates of β-aminopropionitrile-induced AD in ADAMTS-1 knockout mice were significantly lower than those in ADAMTS-1flox/flox mice (Wang S. et al., 2018). ADAMTS-4 has also been shown to be directly associated with AD in a mouse model, causing vascular SMC apoptosis, elastic fiber destruction, and versican degradation in the aortic wall. ADAMTS-4^–/–^ mice had a reduced incidence of AD (Ren et al., 2017). Also, a recent study established that metabolic reprogramming in macrophages has a pivotal role in hypoxia-inducible factor-1α-ADAM17 signaling activation and furthers the development of AD (Lian et al., 2019).

### Effects of ILs on the Aortic Wall

The ILs comprise a family of important proinflammatory cytokines that are thought to be to secreted mainly by macrophages (Shimizu et al., 2006; Golledge et al., 2008). Many ILs participate in the formation of AD. Studies have shown that IL-6, IL-8 (Proietta et al., 2014), IL-11 (Xu Y. et al., 2018), IL-12 (Ye et al., 2018), IL-16 (Fan et al., 2017) and IL-18 (Hu et al., 2019) are elevated in the serum of AD patients, and may represent biomarkers for the diagnosis of AD. Genes related to IL-3 are highly expressed in AD patients, and *in vitro* experiments have shown that IL-3 can increase MMP-12 expression in macrophages via the pathway involving the c-JUN N-terminal kinase and extracellular signal-related kinase 1/2 pathway by binding to the IL-3β receptor (Liu et al., 2018). IL-6 might be required for macrophage activation in the early vascular inflammation that leads to AD (Tieu et al., 2011). Previous studies have shown that IL-6 signaling is mediated by macrophage activation in Ang II-induced vascular disease (Schuett et al., 2009), and that the IL-6-STAT3 model pathway regulates the downstream Th7-IL-17 axis and upregulates monocyte macrophage activity (Ju et al., 2013). IL-18 may promote M1 macrophage differentiation and increase macrophage-induced apoptosis of SMCs (Hu et al., 2019). Taken together, it appears that macrophages secrete ILs to promote local inflammation of the aortic wall, while receiving regulatory signals from ILs to further expand the inflammatory response, for the ultimate promotion of AD.

### VEGF-Mediated Neoangiogenesis Also Contributes to AD Formation

Research from Del Porto et al. (2014) has demonstrated that VEGF-mediated neoangiogenesis plays an important role in ascending aortic wall remodeling. VEGF was mainly found in pro-inflammatory macrophages, and in the endothelial cells that constitute the neovessel walls spreading throughout the tunica media (Del Porto et al., 2014). Neoangiogenesis has been shown to promote inflammation (Reinders et al., 2003) and trigger matrix degradation (Galis et al., 1994) and, therefore, participate in progression and destabilization of atherosclerotic lesions (Slevin et al., 2009). Taken together, it appears that VEGF release and neoangiogenesis may participate in the progression of aortic wall injury, via both inflammation and matrix degradation. In addition, the growth of structurally altered vessels (Melter et al., 2000) that are prone to rupture and bleeding may represent the starting point of the delamination of the aortic media.

In summary, macrophages can secrete a variety of substances involved in AD and related conditions. Macrophages utilize these substances to cause elastic fiber degradation, vascular SMC apoptosis, and neovascularization. This series of changes weakens the aortic wall and creates conditions that are ripe for the development of AD.

## Macrophage-Associated Factors That Protect Against Ad

Substances such as Ang II can regulate macrophages through a series of signal transductions, increasing the incidence of AD. At the same time, there are many protective factors that regulate macrophages in ways that can reduce the incidence of AD.

The suppressor of cytokine signaling 3 (*Socs3*) gene in macrophages may play a critical role in protecting the aorta from AD. In wild-type mice, focal medial disruption of the aorta rarely causes AD development. However, *Socs3* deletion in macrophages increased proliferation and inflammation, biased differentiation of macrophages toward a tissue-destructive phenotype, and dysregulated the differentiation of vascular SMCs. These findings may be clinically relevant, as immunofluorescence staining and imaging cytometry analysis of human AD tissue suggested that adventitial macrophage STAT3 in the aortic wall was activated in regions adjacent to the dissected lesion and at risk of destruction (Ohno-Urabe et al., 2018). Thus, *Socs3* in macrophages appears to act as a protector in stressed aorta, relieving excessive inflammation and triggering the progression of tissue repair, including proper modulation of vascular SMC function.

Glucocorticoids promote vascular remodeling by reducing tumor necrosis factor (TNF)-α secretion and increasing the levels of uncombined soluble TNF receptor II (TNF-sRII) to inhibit AD formation. Increased glucocorticoids reduce the marginalization, extravasation and local activation of macrophages, thereby inhibiting MMP-2 secretion and thus protecting collagen degradation. These findings indicate that glucocorticoids and TNF-sRII may be interesting targets for future AD intervention (Zhang et al., 2018).

Micro RNA (miR)-320 was shown to be significantly downregulated in dissected aortic tissue. MiR-320 may participate in post-transcriptional processing of several MMPs. In fact, overexpressed miR-320 was able to inhibit MMP secretion. A recent study indicated that low miR-320 expression leads to insufficient suppression of MMP secretion, leading to higher expression of MMPs, aggravated ECM destruction, and increased risk of AD (Liao et al., 2018).

Administration of a CD31 agonist peptide greatly reduced the incidence of AD in ApoE^–/–^ mice, and was found bind to wound-associated leukocytes, including macrophages (Fornasa et al., 2012; Andreata et al., 2018). In addition, CD31 signaling in macrophages facilitates aortic remodeling and healing after dissection. An analysis of AD and intramural hematoma samples from patients undergoing surgical treatment revealed that the CD31 expression was lost by the M1 macrophages that densely penetrated into the acute aortic wall lesion sites, with CD31 re-expression accompanied the appearance of M2 and the disappearance of M1 macrophages at the valid aortic wall healing sites (Fornasa et al., 2012; Andreata et al., 2018). M1 macrophages are considered to play a necessary role in early AD proinflammation, and the evidence also supports the view that the transition of wound-related macrophages from the proinflammatory M1 to the pro-reparative M2 phenotype plays a key role in driving inflammation resolution and promoting wound healing (Mantovani et al., 2013; Andreata et al., 2018). These studies suggest that macrophages play an important role in the early stages of AD, and an irreplaceable role in the remodeling and healing of AD. In other words, macrophage activity may persist throughout the course of AD disease.

The macrophage class A1 scavenger receptor (SR-A1)-Tyro3 axis in macrophages reduces AD damage by promoting efferocytosis and suppressing inflammation. SR-A1 deficiency augments AD in mice, facilitates vascular inflammation and apoptosis, and inhibits macrophage efferocytosis. Furthermore, these effects of SR-A1 deficiency can be attenuated by activation of the Tyro3 pathway (Zhang et al., 2019). The beneficial effects of the SR-A1-Tyro3 axis in AD may facilitate the development of a unified mechanism of inflammation and exocytosis in macrophages.

Sestrin-2 (*SESN2*) is an important antioxidant protein that is mainly secreted by macrophages. The expression of Sestrin-2 is significantly higher in the aorta and plasma of AD patients than in healthy donors. In co-cultures of macrophages and SMCs, the overexpression of *SESN2* in macrophages significantly reduced apoptosis of Ang II-induced SMCs; this effect was reversed by *NRF2* silencing. In other words, Sestrin-2 may reduce Ang II-induced SMC apoptosis and participate in AD through the NRF2 pathway (Xiao et al., 2019).

## Conclusion and Outlook

**FIGURE 2:**
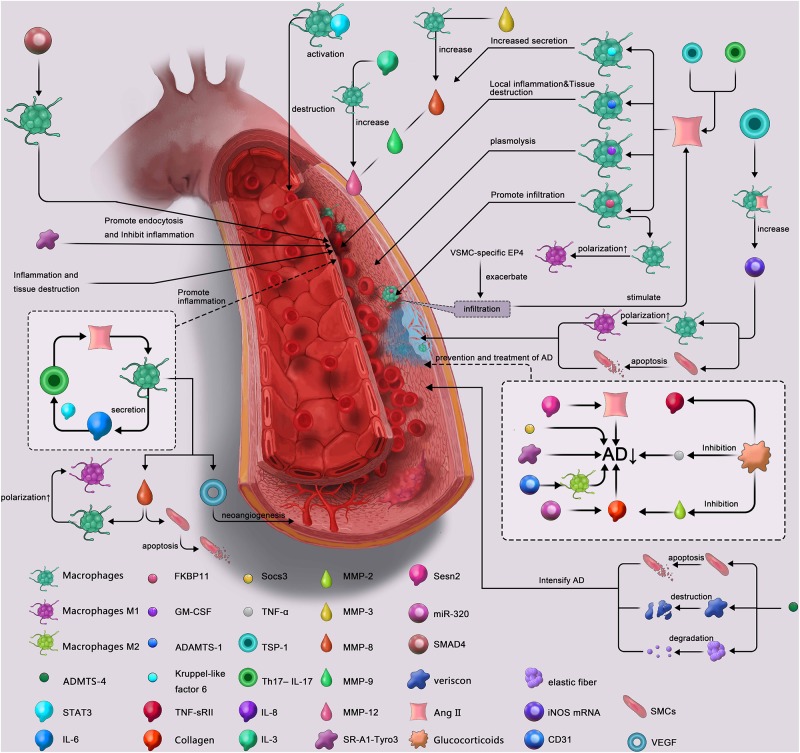
Summarize diagram of the role of macrophage in aortic dissection. AD↓, reduces the incidence of aortic dissection; ADAMTS, a disintegrin and metalloproteinase with thrombospondin motifs; STAT, signal transducer and activator of transcription; IL, Interleukin; GM-CSF, granulocyte macrophage-colony-stimulating factor; TNF-sR II, tumor necrosis factor receptor II; Socs 3, suppressor of cytokine signaling 3; TNF, tumor necrosis factor; TSP-1, thrombospondin 1; Th17-IL-17, T helper cell 17-interleukin -17; MMP, matrix metalloproteinase; SR-A1-Tyro3, A1 scavenger receptor-Tyro3 axis; miRNA, microRNA; Ang II, angiotensin II; iNOS mRNA, inducible nitric oxide synthase mRNA; SMCs, smooth muscle cells; VEGF, vascular endothelial growth factor.

AD is a disease in which the structure of the aortic wall is degraded, eventually causing the intima to rupture, with blood entering the media to form a FL (Han et al., 2018). Macrophages respond mainly to Ang II, and the recruitment, activity and secretion of macrophages in the aortic wall trigger and maintain inflammation there (Dimmeler et al., 1997; Daugherty et al., 2000; Schluter and Wenzel, 2008). Proinflammatory cytokines and metalloproteinases are the main secretions involved in this process. Macrophages have been shown to cause vascular SMC apoptosis, elastic fiber degradation (Ren et al., 2017) and neovascularization (Del Porto et al., 2014) leading to the destruction and separation of the aortic wall in both humans and mice. M2 polarization also plays an important role in the repair and remodeling process after the dissection has occurred, with M2 macrophages inhibiting the development of inflammation and promoting the repair of local tissues (Fornasa et al., 2012; Mantovani et al., 2013). This feature is critical to the stability of AD. In general, macrophages play a crucial role in AD and are potential targets for the prevention and treatment of this disease. And, the summarize diagram of this review was shown in Figure 2.

Increasingly, AD-related studies are shifting their focus to aortic inflammation and the role of macrophages in AD formation and repair (Fornasa et al., 2012; Andreata et al., 2018). Thus far, efforts at understanding the contribution of inflammation and macrophages to AD have relied upon analyses of clinical samples and mechanistic studies in animal models. However, because of the contingency of lethality of AD disease, it is rare to obtain preoperative samples or imaging data in patients, and most experiments in animals are designed to wait for the AD to occur before material is collected for analysis. Monitoring of the dynamic changes in inflammation and macrophage activity throughout the development of AD would allow us to distinguish between the two possibilities of accumulated inflammation that eventually leads to AD, and intense inflammation that is triggered by AD. Currently, there is only one medical case report with documented abnormal inflammatory activity in the aortic wall that gradually increased in the 5 years leading up to the patient’s eventual AD (Tahara et al., 2016). Because the patient in that case received repeated combined positron emission tomography (PET)/computed tomography, it is possible that technologies such as PET and 4D magnetic resonance imaging could play a crucial role in revealing the mechanisms of AD, dynamic changes in inflammation and macrophage activity.

## Author Contributions

HZ contributed in interpretation of data of the work and critical revision of the work. AM contributed in analysis of data of the work, figure drawing, and critical revision of the work. All authors above have approved the version to be published and agreed to be accountable for all aspects of the work in ensuring that questions related to the accuracy or integrity of any part of the work are appropriately investigated and resolved.

## Conflict of Interest

The authors declare that the research was conducted in the absence of any commercial or financial relationships that could be construed as a potential conflict of interest.
